# Tissue Engineering Approaches in the Design of Healthy and Pathological *In Vitro* Tissue Models

**DOI:** 10.3389/fbioe.2017.00040

**Published:** 2017-07-26

**Authors:** Silvia Caddeo, Monica Boffito, Susanna Sartori

**Affiliations:** ^1^Department of Mechanical and Aerospace Engineering, Politecnico di Torino, Turin, Italy; ^2^Department of Oral Cell Biology, Academic Center for Dentistry Amsterdam, Amsterdam, Netherlands

**Keywords:** bone, heart, liver, models, pancreas, three-dimensional, tissue engineering

## Abstract

In the tissue engineering (TE) paradigm, engineering and life sciences tools are combined to develop bioartificial substitutes for organs and tissues, which can in turn be applied in regenerative medicine, pharmaceutical, diagnostic, and basic research to elucidate fundamental aspects of cell functions *in vivo* or to identify mechanisms involved in aging processes and disease onset and progression. The complex three-dimensional (3D) microenvironment in which cells are organized *in vivo* allows the interaction between different cell types and between cells and the extracellular matrix, the composition of which varies as a function of the tissue, the degree of maturation, and health conditions. In this context, 3D *in vitro* models can more realistically reproduce a tissue or organ than two-dimensional (2D) models. Moreover, they can overcome the limitations of animal models and reduce the need for *in vivo* tests, according to the “3Rs” guiding principles for a more ethical research. The design of 3D engineered tissue models is currently in its development stage, showing high potential in overcoming the limitations of already available models. However, many issues are still opened, concerning the identification of the optimal scaffold-forming materials, cell source and biofabrication technology, and the best cell culture conditions (biochemical and physical cues) to finely replicate the native tissue and the surrounding environment. In the near future, 3D tissue-engineered models are expected to become useful tools in the preliminary testing and screening of drugs and therapies and in the investigation of the molecular mechanisms underpinning disease onset and progression. In this review, the application of TE principles to the design of *in vitro* 3D models will be surveyed, with a focus on the strengths and weaknesses of this emerging approach. In addition, a brief overview on the development of *in vitro* models of healthy and pathological bone, heart, pancreas, and liver will be presented.

## Introduction

Tissue engineering (TE) was defined by Langer and Vacanti in early 90s as “an interdisciplinary field which applies the principles of engineering and life sciences toward the development of biological substitutes that restore, maintain, or improve tissue function” (Langer and Vacanti, 1993). TE aims to induce tissue-specific regeneration processes, thus overcoming the well-known drawbacks of organ transplantation (i.e., donor shortage, need of immunosuppressive therapy). TE approaches have been recently proposed for the design of reliable *in vitro* models of healthy or pathological tissues and organs, which can be employed for drug screening and the evaluation of new therapies, as well as the investigation of the complex phenomena regulating disease onset and progression. Besides their high scientific potential, these models also bring some advantages in terms of ethical and economic issues.

From the ethical point of view, the employment of animals for biomedical research purposes has been thoroughly debated and the topic still opens the door to discussion (Festing, 2004; Pound and Bracken, 2014). The principle of 3Rs (Replacement, Reduction, and Refinement) introduced by Russell et al. (1959), which encourages the research community to recognize the importance of welfare for animals used in science, is currently embedded in national and international legislation. In view of this, a large amount of resources have been invested to develop methods to replace animals in research. Moreover, although animal models have significantly contributed to both our understanding of human biology and the development of modern medicine (Festing, 2004), they often show limits in the reproduction of specific human conditions (Dixit and Boelsterli, 2007). Even though some human pathologies can be induced in animal models, the molecular mechanisms driving their onset and progression are often significantly different (Dixit and Boelsterli, 2007; Pound and Bracken, 2014). The increasing number of existing animal models and the inefficacy on humans of some drugs successfully tested on animals are symptoms of animal model inability to effectively recapitulate human physiology.

Economic aspects should be also considered: the actual costs for successfully transforming a drug candidate from a new molecular entity (NME) to a clinical product are between $800 million and $2.2 billion, with development timelines spanning 8–12 years (DiMasi et al., 2016). Moreover, there is a high failure rate for NMEs in lead development, especially those in expensive late-stage clinical trials. It has long been recognized that two-dimensional (2D) cell monocultures used in preclinical studies lack many of the requisite phenotypic characteristic often necessary for their utility in predictive drug assays (Grainger, 2014). The three-dimensional (3D) environment in which cells grow *in vivo*, in fact, allows them to actively interact with the surrounding extracellular matrix (ECM) and cells, thus providing stimuli (e.g., soluble factors and physical forces) that strongly influence their functions and gene expression profile. Moreover, the accumulation of waste products in the culture medium, the limited nutrient supply, and the lack of cell-specific mechanostimulation often result in cell death or loss of functions, as a consequence of the non-physiological cell culture conditions (Mosig, 2016).

In order to overcome the above mentioned limitations of current drug-screening methodologies and reduce the use of animals, 3D models have been investigated and a number of studies are in progress, with the aim of making them more and more reliable and sophisticated. Furthermore, unlike animal models, 3D *in vitro* models give the possibility to independently identify and modulate cellular and molecular factors responsible for disease onset and progression, allowing the investigation of the contribution of each of them on the development of a specific disease and thus changing the way to study tissue physiology and pathophysiology. The introduction of these models in the biomedical research practice may lead to numerous advantages, such as the reduction of animal use as well as the overcoming of the limits associated with traditionally employed models (i.e., animal and 2D cell culture models), and the achievement of more reproducible data, thanks to the possibility to tightly control the experimental parameters, with lower cost and less time. A 3D *in vitro* model allows the cells to grow and interact with each other and with the ECM in the all spatial dimensions. The 3D structure can be achieved through a 3D matrix support (scaffold) or by using scaffold-free organoids cultures. Fatehullah et al. (2016) have recently published a comprehensive review on 3D *in vitro* models based on organoids. In this review, we first give a brief overview on the main components of an *in vitro* 3D tissue-engineered model that should recreate *in vitro* the *in vivo* surrounding environment and stimuli, i.e., the 3D porous matrix (scaffold), the cells, and the applied cues (biochemical or physical). Then, we discuss how the TE approaches have been employed in the modeling of two of the most studied engineered tissues, bone and cardiac tissue, as well as some less engineered organs, which are of considerable interest due to the incidence of their pathologies, namely pancreas and liver.

## Scaffolds, Cells, and Stimuli for *In Vitro* Modeling

**Figure 1:**
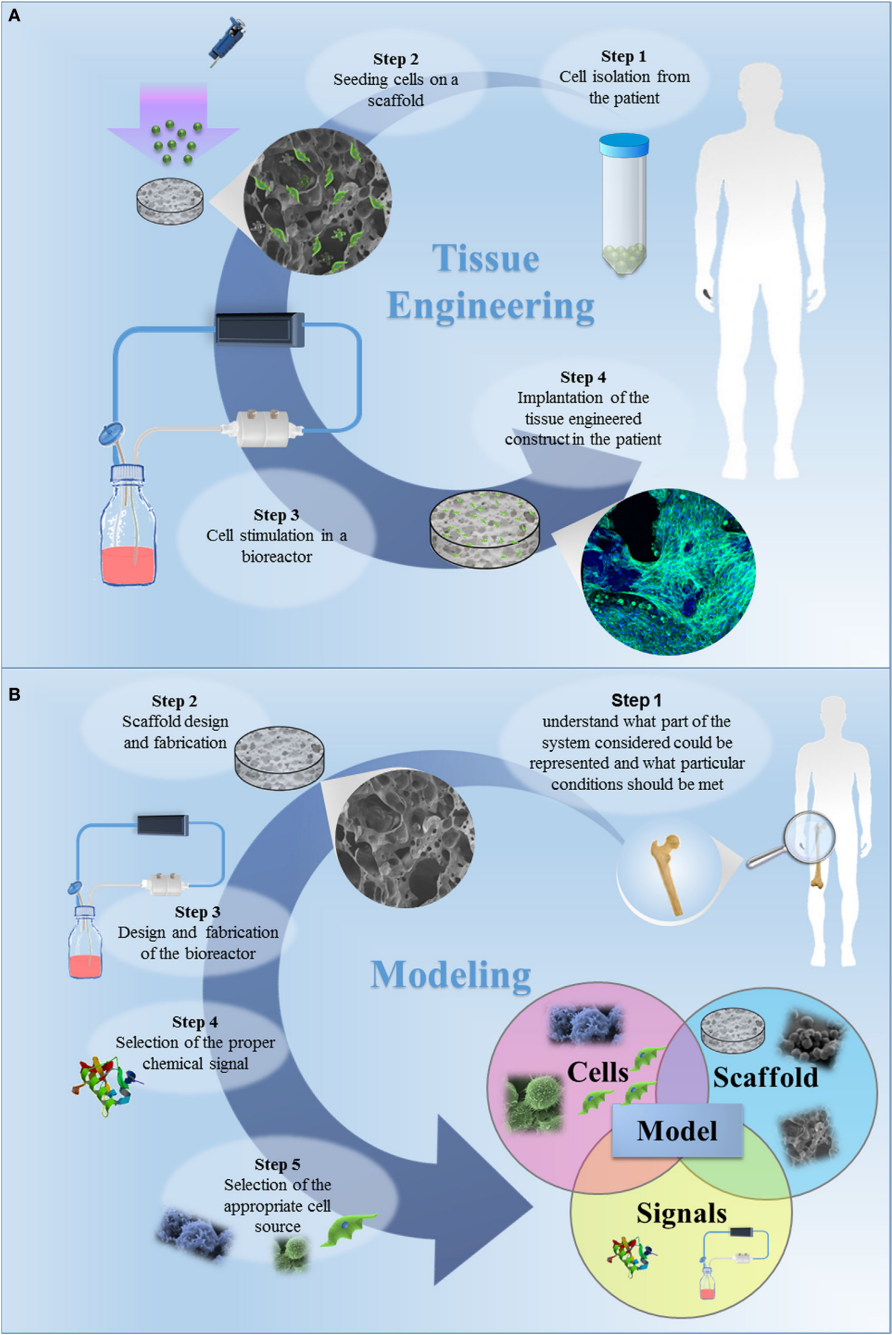
**(A)** The classic tissue engineering (TE) paradigm consists in the isolation of primary cell from the patient and the further seeding on a three-dimensional (3D) porous scaffold, specifically designed to induce cell proliferation and differentiation. The bioartificial system is developed in a specific environment, where the metabolic, mechanical and electrical stimuli are provided by a bioreactor. In this tissue-specific designed system, cells start to produce extracellular matrix, leading to *in vitro* tissue maturation. The tissue-engineered construct is then implanted in the patient, undergoing to a remodeling process *in vivo*, which allows the tissue restoration. **(B)** The TE approach for the development of *in vitro* models. The first step is to understand what part of the system considered should be represented and which specific conditions should be met. The second step is the design of the appropriate scaffold, i.e., the design of a 3D porous construct recapitulating the architecture and the mechanical and surface properties of the tissue/organ to be modeled. The third step is the selection of the cells to be seeded in the designed scaffold. The subsequent steps should be the design and fabrication of a bioreactor and the selection of the appropriate chemical signals (e.g., cocktail of growth factors) to be included in the model. Once a model is successfully developed, it can be improved by increasing its complexity, by introducing additional cell type, chemical factor or coupling different mechanical stimuli. The final step is the validation, aiming to demonstrate the relevance of the model for the intended purpose. The validation procedure is fundamental and this step should be carefully designed.

The classic TE paradigm involves the combination of living cells with a natural, synthetic, or bioartificial support to develop a biological substitute or a 3D living construct that is structurally, mechanically, and functionally equal to a tissue (Figure 1A) (Kim and Evans, 2005). Moving from the first definition of TE in the early 90s through the huge amount of work carried out and published by many research groups all over the world, researchers have gained high expertise in cell manipulation, materials science, and bioengineering for the design of highly complex biomimetic tissue substitutes for reparative/regenerative purposes. These tools and specific competences have been transferred in the last decade to the development of 3D engineered *in vitro* models, as schematized in Figure 1B. In its current definition, modeling does not mean to finely replicate *in vitro* the native tissue/organ, but that the model should be properly designed to recapitulate the particular conditions that are intended to be mimicked. Effectively mimicking a tissue is extremely complex since several aspects must be taken into account and each single tissue has different features. In this context, the fundamental elements that should be considered are (I) scaffolds, (II) cell sources, and (III) chemical and physical stimuli.

### Scaffolds

The complexity of *in vivo* tissue organization allows cells to interact with each other and with the surrounding ECM. In an engineered *in vitro* model, the scaffold must be designed to finely replicate *in vitro* the architecture of the native tissue, i.e., its ECM framework to let cells to adhere, spread, proliferate, differentiate, maturate, and produce ECM, similarly to what they do *in vivo*.

The combination of competences among materials science, biomedical engineering, and molecular biology has allowed the complex interaction between cell and materials to be better understood (Causa et al., 2007). The choice of the most suitable biomaterial for scaffold fabrication is a key element for the model design, since it strongly influences cellular functions. Biomaterials should be carefully selected depending on the modeled tissue/organ, acting as a synthetic ECM that interacts with cells at the molecular level, influencing cell functions and driving the complex cellular processes that lead to the development of a valid *in vitro* engineered tissue model. Material selection strongly depends on tissue mechanical properties, since the scaffold’s mechanical properties should match those of the tissue to be modeled, in healthy and pathological conditions. In fact, a pathologic tissue presents altered ECM properties, e.g., the different architecture and mechanical properties of osteoporotic bone compared to its healthy counterpart (Chen et al., 2010), and tissues stiffening under inflammatory conditions and aging processes, as discussed in more detail for pancreas and cardiac tissue in the following paragraphs (Ziol et al., 2005; Brower et al., 2006; Georges et al., 2007; Kwak, 2013). Thus, to model a pathologic system, the scaffold should be designed in order to reproduce these altered ECM features. A straightforward approach could consists in mimicking the dynamic of disease progression through the employment of scaffolds with time-varying properties. For instance, hydrogels can be properly designed so as to present time-dependent stiffening by directing their crosslinking reaction (Young and Engler, 2011; Guvendiren and Burdick, 2012).

Mechanobiology studies have highlighted the importance of scaffold mechanical properties to properly direct cell behavior (Chan and Leong, 2008). Due to their high stiffness and load-bearing properties, ceramics and their composites are generally used in TE of hard tissues, while polymers are mainly employed in the engineering of soft tissues (Seidi and Ramalingam, 2012).

Material surface also plays a key role in guiding cell behavior and fate, being the primary interface for cell interaction. In order to obtain the desired cell response, it is possible to modify the biomaterial surface with bioactive molecules, such as specific proteins or peptide sequences (e.g., the fibronectin-derived arginylglycylaspartic acid peptide sequence -RGD-), that are recognized by cells as integrin-binding domains. Typically, surface modification takes place after scaffold fabrication, without affecting its structure or mechanical properties (Ma, 2008). The characteristics of the final model strongly depend on the selected fabrication technique and the possibility to tailor the processing parameters in order to fulfill the requirements for the intended application. Thus, TE scaffolding techniques developed for regenerative medicine can be exploited for modeling applications. Both the top-down and the bottom-up approaches have been employed for the design of tissue-engineered *in vitro* models. In top-down strategies, cells are cultured on scaffolds specifically designed to mimic the tissue to be modeled in terms of structure, composition, and mechanical properties. On the other hand, the bottom-up approach aims to mimic and replicate the functional unit of a tissue and to create a more biomimetic scaffold. These modular scaffolds can be obtained through both microencapsulation and microfabrication techniques as well as employing traditional cell culture strategies (Nichol and Khademhosseini, 2009).

The scaffold should present a high degree of porosity, with interconnected pores and pore dimension adequate for the specific application. This porous architecture allows cells migration also in the inner part of the 3D construct and permits nutrient/oxygen diffusion and waste removal (Causa et al., 2007). Several research works demonstrated that the morphology of cells cultured on 3D scaffolds significantly differs from that of cells cultured on 2D surfaces (Hamilton et al., 2006; Miyagawa et al., 2010). Most cells are able to differentiate and develop a physiologically relevant tissue *in vitro* only if cultured in a 3D environment (Griffith and Swartz, 2006; Khetani and Bhatia, 2006; Pampaloni et al., 2009). Moreover, 3D patterned scaffolds have shown to promote greater cell aggregation, proliferation, and differentiation than 2D substrates (Hamilton et al., 2006; Lee et al., 2009; Sato et al., 2010; Eniwumide et al., 2014). The pattern size influences cell morphology, proliferation, and migration (Zong et al., 2005; Kai et al., 2011; Sunami et al., 2014). It was also demonstrated that cell response to the substrate morphology strongly depends on the cell type (Chang and Wang, 2011). Topography can also enhance the differentiation of progenitor cells into their programmed pathway (Yim et al., 2007). All of these observations show the importance of creating appropriate microstructures able to mimic the native tissue. To replicate the spatial gradient of properties, composition, and functions that is typical of many biological tissues (e.g., bone and cartilage), scaffolds with functional spatially distributed gradients have been developed (Leong et al., 2008). The design of these scaffolds is complex and requires the employment of computer-aided tools and computational modeling to guarantee a biomimetic environment for *in vitro* tissue development (Mattei et al., 2012; Mattei and Vozzi, 2016).

### Cell Sources

The choice of the most appropriate cell source is a challenge in the design and further development of a tissue-engineered model. In fact, the development of representative *in vitro* tissue/organ models depends on the availability of tissue-specific cellular phenotypes, able to recapitulate *in vitro* the characteristics of normal or pathological natural tissues. Moreover, the number of cells to be included in the model should be carefully considered to guarantee a physiologically relevant 3D replica of the tissue functional unit (Mattei et al., 2014). To bridge the gap between animal models and clinical trials, *in vitro* models should include human cells (Griffith and Swartz, 2006; Pampaloni et al., 2009; Maltman and Przyborski, 2010). Most of the human cells employed in TE are adult primary cells isolated from patients. These cells are representative of the functional unit of a tissue, since they can be isolated from tissue biopsies harvested from healthy or pathological patients. On the other hand, adult primary cells have limited life span and low proliferation rate, and their isolation procedure is complex (Benam et al., 2015). To overcome these limits, stem cells have been employed. Stem cells are undifferentiated cells able to both self-renew and differentiate to one or more types of specialized cells, which can be isolated from different sources, such as embryos, fetuses, or adult tissues, and their differentiation capability depends on the cell source. The main critical issues in stem cell employment concern (i) the ability to control cell differentiation pathways toward the desired lineages and (ii) the immature phenotype of the cells derived from stem cells, which have a gene expression profile similar to fetal cells. Nevertheless, in the design of new *in vitro* systems for pharmacological and toxicological tests, the use of human stem cells represents a fundamental resource. As an example, cardiomyocytes differentiated from human embryonic stem cells (ESCs) have been successfully employed as pharmacological model for the evaluation of different cardioactive drugs (Harding et al., 2007). Induced pluripotent stem cells (iPSCs) are engineered stem cells derived from differentiated somatic cells by overexpression of specific transcription factors (Takahashi et al., 2007; Yu et al., 2007). These cells have characteristics similar to pluripotent ESCs and, under certain conditions, can differentiate toward various phenotypes. Moreover, iPSCs can be isolated from patients affected from a specific pathology, thus allowing the *in vitro* modeling of the disease and the study of the mechanisms involved in its onset and progression. Patient-derived iPSCs lines can be also exploited as cellular assays for new drug testing and safety assessments, opening the way to a personalized method that can vary in function of the patient/pathology (Szebényi et al., 2011). The relevance of iPSCs *in vitro* disease modeling has been recently reviewed by Benam et al. (2015).

### Physicochemical Stimuli

The *in vivo* environment guarantees the presence of fundamental molecular cues that direct cell behavior, while the vascularization provides nutrient supply and waste removal. Thus, the presence of molecular factors influencing cellular division, shape, spreading, proliferation, death, and secretion of ECM components is necessary to successfully model morphogenetic events (Gilbert, 2000).

In the design of a 3D model, it should be considered that cells in the middle of the construct could behave differently from cells growing on the surface, depending on nutrients concentration gradient. The development of a successful 3D engineered tissue could be hindered by a limited diffusive transport of nutrients through its thickness. To avoid local concentrations and overcome the diffusion limits, that affect cell behavior, chemical, and mechanical signals should be coupled (Griffith and Swartz, 2006). Moreover, cells are subjected to extracellular and intracellular mechanical forces *in vivo* that determine their fate. In particular, cells respond to dynamic cues, such as electric fields, osmotic and hydrostatic pressure, stress, strain, fluid flow, and streaming potential, by modifying the surrounding ECM (Raimondi, 2006). Mechanical stimuli are usually provided to tissue-engineered constructs by bioreactors specifically designed to reproduce the *in vivo* conditions.

In particular, bioreactors provide mechanical or electrical stimuli and allow a fine modulation of culture conditions to reach tissue maturation (Martin et al., 2004; Massai et al., 2013). Microscale technologies, such as novel platforms based on microfabrication and microfluidics, have shown to be another important tool, allowing real-time monitoring and high-throughput results, with the possibility to test a single parameter in an independent way (Rouwkema et al., 2011). These technological devices could be a valid support for the development of 3D models with relevant functional characteristics, assuring a good reproducibility. The reviews published by Ghaemmaghami et al., Huh et al., and Inamdar and Borenstein provide some insights on this topic (Huh et al., 2011; Inamdar and Borenstein, 2011; Ghaemmaghami et al., 2012).

## Bone *In Vitro* Models

Bone is a connective tissue in which cells are surrounded by an ECM constituted by an inorganic phase (~70% w/w) and an organic phase (~30% w/w) (Boskey, 2004; Alvarez and Nakajima, 2009). The inorganic phase is mainly composed by hydroxyapatite, while the organic one is constituted of type I collagen and other non-collagenous proteins. Bone ECM 3D organization allows the transmission of mechanical stresses, which have shown to be fundamental for bone development (Carter et al., 1996). Bone homeostasis is normally guaranteed by the synergic action of osteoblasts and osteoclasts, which respectively secret and resorb bone ECM (Buckwalter et al., 1995) in the bone remodeling process. More than 90% of the bone cells population is constituted by osteocytes that are responsible for sensing and transducing the mechanical forces transmitted through the bone and consequently orchestrating the signals of bone resorption and deposition (Franz-Odendaal et al., 2006).

Cell cultures on classic plastic 2D supports and animal models have been largely employed to study the basic mechanisms driving bone physiological and pathological processes, but they have been shown to be limited in many applications. Traditional 2D culture lacks the capability to imitate the 3D microenvironment of the native bone, which is fundamental for the regulation of cell–cell and cell–ECM interactions (Tortelli and Cancedda, 2009). One of the main limitations of animal models employed for orthopedic research is the interspecies variation of bone tissue. It has been demonstrated that bone composition, density, and mechanical properties of commonly employed animal models (i.e., cow, sheep, pig, dog, chicken, and rat) are significantly different from those of humans (Aerssens et al., 1998). In particular, the rat, which is the most commonly employed animal model (Martini et al., 2001), presents the most significant differences from human bone (Aerssens et al., 1998). Moreover, animal models employed to study bone physiopathology present limitations due to differences in bone healing and remodeling processes (Mills and Simpson, 2012). One of the most common human bone diseases is osteoporosis: it has been recently estimated that one in five men and one in three women over 50 will experience an osteoporotic fracture in their lifetime (www.rsc.org/education/eic/issues/2006Nov/GlassBones.asp). This pathology derives from an imbalance in the remodeling process in which bone resorption prevails on bone deposition and it has high prevalence in postmenopausal women (Jabbar et al., 2011). Animal models for the study of osteoporosis have been thoroughly reviewed in the literature in the last decades (Jee and Yao, 2001; Turner, 2001; Turner et al., 2001; Egermann et al., 2005; Komori, 2015). It has to be noted that only human and non-human primates may naturally be affected by osteoporosis (Cerroni et al., 2000). However, acquisition of aged female primates is difficult, expensive, and risky in term of zoonotic diseases transmission (Turner, 2001). The most common approach is the induction of osteoporosis in animals through ovariectomy. However, none of the already available animal models is able to accurately reproduce the conditions of postmenopausal osteoporosis (Egermann et al., 2005). Therefore, several different models are usually employed at the same time (Thompson et al., 1995), with an increase in costs, without the assurance of reaching predictable results of what occurs in humans.

Alternative 3D models have been proposed to reproduce *in vitro* the bone environment. The first example of *in vitro* bone model is represented by explants of bone tissue cultured *ex vivo*, closely mimicking the *in vivo* situation, with the main advantage of isolating local effects from systemic factors (Sajeda et al., 1998). *Ex vivo* cultured bone models have been employed for the study of bone cell biology and the interactions of cells with each other and with the surrounding bone ECM. Nevertheless, the limited nutrient and oxygen supply to the central portion of the explanted bone cultured under static conditions leads to cell necrosis, constituting a big limitation of *ex vivo* models (Simpson et al., 2009). The introduction of bioreactors allowed an increase in cell viability and the development of long-term *ex vivo* cultured models, demonstrating the importance of mechanical stress on osteoblast behavior (David et al., 2008; Simpson et al., 2009). However, the use of explants of human bone tissue cultured *ex vivo* has shown a large variability due to the different sex, age, health conditions, and life style of the donor patients (Simpson et al., 2009).

In the context of modeling, where reproducibility plays a key role, the employment of TE models based on cells cultured on 3D porous scaffolds looks like a valid alternative.

To the best of our knowledge, the first *in vitro* TE-based 3D model of bone turnover was developed by Tortelli et al. (2009). The molecular mechanisms driving bone turnover was investigated by coculturing murine primary osteoblast and osteoclast precursors for 40 days on a porous ceramic scaffold (Skelite^®^). In this 3D model, osteoclasts showed the tendency to differentiate when osteoblasts were already differentiated, but the ECM was not completely organized. Under osteogenic stimulation, enhanced bone deposition and reduced resorption were observed, because of the increased expression from osteoblasts of osteoprotegerin, a natural inhibitor of osteoclast formation and functions (Kular et al., 2012). This model demonstrated that the 3D environment is able to stimulate osteoprogenitor differentiation in osteoblasts, which in turn promotes osteoclast precursor differentiation in osteoclasts. However, the use of animal cells in this model did not permit to overcome the limits associated with the use of animal-based models. Thus, the use of human cells is required to develop a relevant alternative model.

Few years later, Papadimitropoulos et al. (2011) proposed a bone tissue model for the study of bone turnover based on the coculture of osteoblastic/osteoclastic human cells and endothelial cells. A 3D porous ceramic scaffold composed of hydroxyapatite and beta-tricalcium phosphate was first seeded with osteoprogenitors and endothelial cells, isolated from the stromal vascular fraction of human adipose tissue and then with osteoclast progenitors derived from human peripheral blood. The cells were cultured in a perfusion bioreactor in the presence of osteoclastogenic factors (κB factor ligand, dihydroxyvitamin D_3_). This study demonstrated that the osteoclastic resorption activity is strictly correlated to the presence of pre-seeded osteoprogenitors that synthetize ECM components. This 3D multi-culture construct was the first model coupling osteoclastic matrix resorption to active matrix deposition by osteoblastic cells. Once validated, such a model may find application in the testing of drugs regulating bone turnover, or in the evaluation of new bone substitute biomaterials, reducing the use of laboratory animals, according to the 3Rs principle (Russell et al., 1959).

A more complex *in vitro* model of the bone remodeling process was recently developed by Bongio et al. (2016), culturing human osteoblast and osteoclast precursors with human umbilical endothelial cells (HUVECs) and including human bone marrow-derived mesenchymal stem cells (HBMSCs). In fact, HBMSCs cocultured with endothelial cells can differentiate in mural cells (pericytes and vascular smooth muscle cells), which have a key role in the development and maintenance of the vascular network (Armulik et al., 2005). The cells were tetra-cultured in a collagen/fibrinogen hydrogel enriched with calcium phosphate nanoparticles for 10 days. The developed 3D construct showed a good *in vitro* vascularization and improved osteoclast and osteoblast differentiation compared to the respective monocultures. HUVEC and HBMSCs positively influenced the differentiation processes, with enhanced differentiation of both osteoclasts and osteoblasts in the tetra-culture compared to their coculture. This research provides another example of successfully coupling *in vitro* the osteoblast-mediated mineralization (bone deposition) and osteoclast digestion of calcium phosphate (bone resorption). The authors also anticipated that the developed model could be finely tailored to mimic the remodeling process in the presence of pathologies correlated to bone homeostasis dysregulation (e.g., osteoporosis and sclerosteosis). However, it has to be demonstrated first that the cell functions can be preserved in the construct for long-term cultures in order to constitute a valid *in vitro* model. The main limitation of this model is the lack of imitation of bone ECM characteristics, being scaffold stiffness and architecture (i.e., porosity, pore size, distribution, and interconnection degree) important cues to direct osteoblast precursor differentiation toward the osteogenic lineage (Engler et al., 2006) and mimic the native bone tissue and its health state.

Although osteocytes have been shown to play an important role in regulating osteoblast activity (Taylor et al., 2007), the aforementioned studies did not include these cells in the models. Since human primary osteocytes do not proliferate *in vitro*, most of the research around this cell type is performed employing murine osteocyte-like cell lines (MLO-Y4, MLO-A5, IDG-SW3, and Ocy454) (Wittkowske et al., 2016). The large majority of the *in vitro* models employed to study osteocyte functions are surprisingly 2D, not mimicking the *in vivo* physiological conditions where osteocytes are embedded in the ECM. In a 3D model developed by Vazquez et al. (2014), MLO-Y4 cells showed an osteocyte-like morphology and the formation of a 3D network. In this study, MLO-Y4 were embedded in a type I collagen-based matrix and cocultured for 1 week with human osteoblast-like cells (MC3T3-E1 or MG63) to investigate cell response under mechanical stimulation (Vazquez et al., 2014). This model could constitute a valid instrument to study the interactions between osteoblasts and osteocytes, but its main drawback concerns the use of cell lines instead of human primary cells.

**Figure 2:**
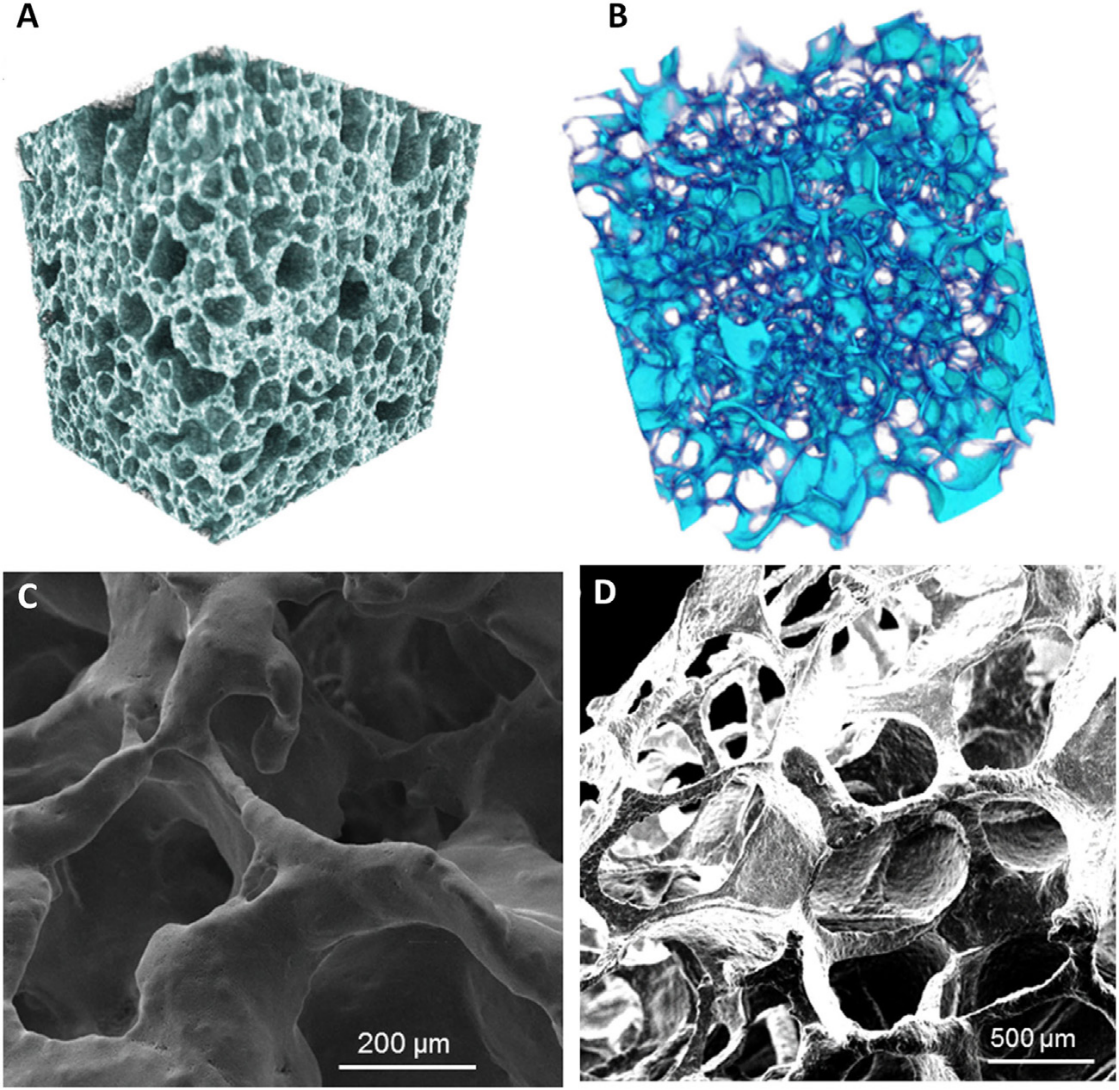
Micro-CT reconstruction of an example of glass–ceramic scaffolds mimicking **(A)** healthy bone and **(B)** osteoporotic bone architectures. SEM micrographs of **(C)** a “healthy” scaffold and **(D)** an “osteoporotic” scaffold. Magnifications: **(C)** 300×, **(D)** 100×. Reprinted with permission from Baino et al. (2016).

The importance to develop tissue-engineered pathology-specific bone models has been recently highlighted by Baino et al. (2016). Actually, they developed 3D bioceramic scaffolds mimicking the morphology and architecture of normal and osteoporotic bone trabecular tissues (Figure 2), with the aim to overcome the lack of suitable models to study osteoporosis. The scaffolds were fabricated using a bioactive glass, CEL2, previously designed by Vitale-Brovarone et al. (2007). They successfully employed the sponge replication technique to finely tailor the structure of the resulting 3D scaffold. In particular, they studied the effect that the processing parameters have on the final scaffold properties by varying the sponge template porosity, the slurry composition (made mainly from water, polyvinyl alcohol and CEL2 powder), the number of impregnation steps of the template in the slurry, and the final compression step to remove the excess of slurry. The obtained scaffolds showed total porosity, pore size, and strut thickness in the range of those of healthy (Hildebrand et al., 1999) and pathological (Chiba et al., 2013) trabecular human bone of the femoral head. This research could open the door to new modeling approaches for the *in vitro* investigation of osteoporosis, once the scaffold will be seeded with relevant cells in order to obtain a complete tissue-engineered model.

The TE approach was also exploited by Matziolis et al. (2006) for the development of an *in vitro* model of the early phase of bone fracture healing, which aimed to support preclinical testing of novel therapeutic approaches. In order to mimic the neighboring bone fragments at the interface with the fracture hematoma, the scaffold was made from two discs of lyophilized human cancellous bone with a fibrin suspension in the middle. The cells selected for the model were rabbit periosteal osteoprogenitor cells and the final construct was cultured for 2 weeks under dynamic conditions in a bioreactor, with the aim to simulate the *in vivo* mechanical stimulation. In particular, the bioreactor was specifically designed to allow the mimesis of different conditions, such as partial or full weight-bearing and normal walking, by applying cyclic pneumatically driven compression stresses on silicone membranes. The major limitation of the model was the absence of immunocompetent cells, such as platelets, polymorphonuclear neutrophils, monocytes, and macrophages (Bueno and Glowacki, 2011), responsible for the inflammatory response that constitutes the initial phase of fracture healing (McKibbin, 1978). Moreover, the model lacked growth factors and cytokines that are commonly released after traumatic events. Periosteal cells embedded in the fibrin matrix responded to the applied stimulation by producing type IX collagen, thus demonstrating endochondral ossification and showing similarities to the early phase of fracture repair.

As an alternative to human bone primary cells that are highly sensitive to culture conditions, source, patient age, and isolation/purification techniques, and lost differentiation ability after a low number of passages in culture (Bouet et al., 2014), primary mesenchymal stem cells (MSCs) can be isolated from bone marrow, adipose tissue or periosteum, and can be induced to differentiate toward the osteogenic lineage. Another alternative can be represented by the use of human iPSCs (hiPSCs), which have been recently employed in bone TE for regenerative applications (TheinHan et al., 2013; Kang et al., 2014; Tang et al., 2014; Jeon et al., 2016; Ji et al., 2016). These cells may constitute an interesting cell source for the design of *in vitro* models of healthy and pathologic bone tissue. A complete 3D *in vitro* bone model including human osteoblasts, osteoclasts and osteocytes has not been developed yet. The combination of all bone cells in an *in vitro* bone model is challenging and will be of utmost importance to better understand bone cell biology, i.e., to elucidate the role of osteocytes in bone remodeling.

## *In Vitro* Cardiac Tissue Models

The heart is a complex organ containing a wide variety of cell types (e.g., cardiomyocytes, Purkinje cells, fibroblasts) embedded in an anisotropic and hierarchical architecture. The activity of heart cells is regulated by an internal control system, sensitive to both external and systemic cues (Benam et al., 2015). Such a complex architecture is supported by a well-defined 3D framework based on fibrous proteins (e.g., collagen, elastin), adhesive glycoproteins (e.g., laminin, fibronectin), and proteoglycans (Parker and Ingber, 2007). This 3D ECM network is responsible for the typical organization of cardiac muscle fibers along a preferred direction, which results in the peculiar mechanical properties and cell functions characterizing the myocardium.

**Figure 3:**
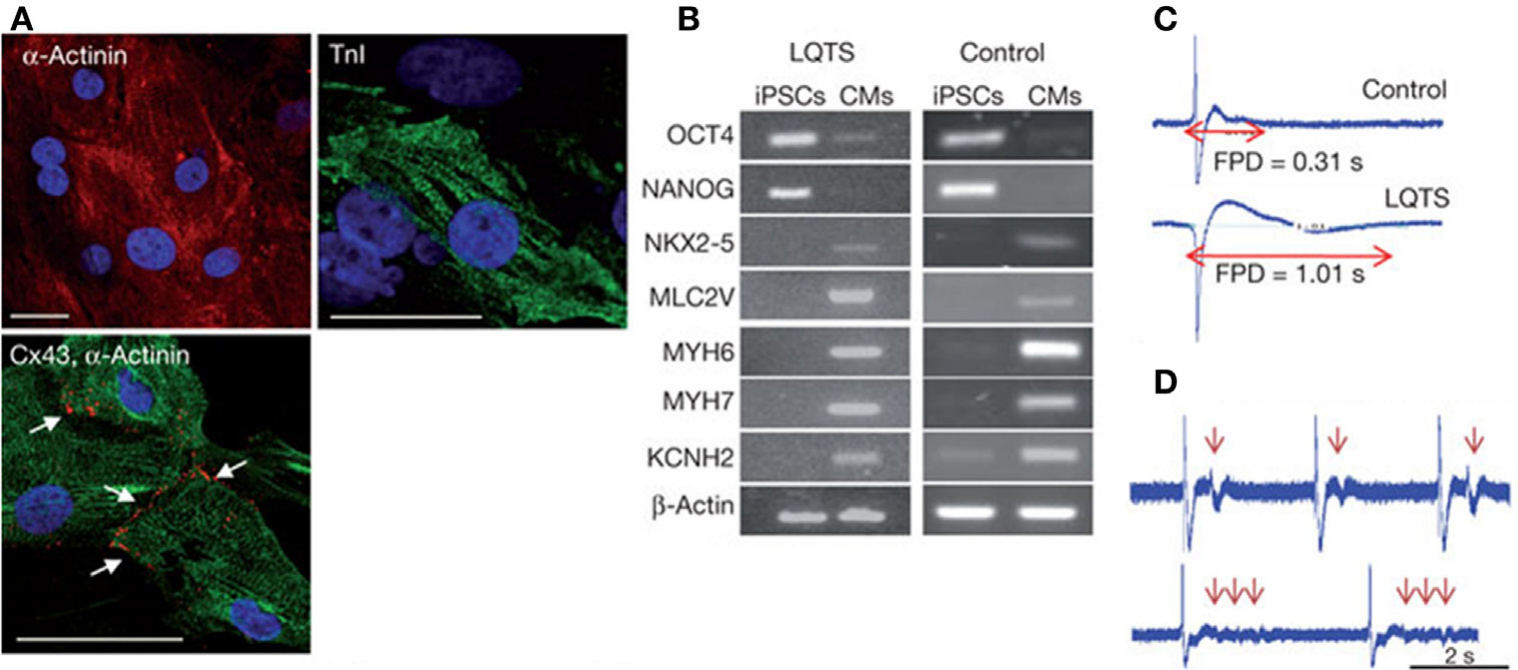
Human iPSCs (hiPSCs) were obtained by reprogramming dermal fibroblasts. Upon differentiation toward the cardiac phenotype (hiPSC-CMs), the two-dimensional cardiac tissue model was designed by plating the cells onto fibronectin-coated glass coverslips. **(A)** hiPSC-CMs suffering long QT syndrome (LQTS) stained for α-actinin, troponin I (TnI) and connexion 43 (Cx43). White arrows identify gap junctions Scale bars: 30 µm. **(B)** Polymerase chain reaction results showing that both LQTS and control hiPSC-CMs express cardiac-specific transcription factors (NKX2-5), sarcomeric proteins (MYH6, MYH7, MLC2V), and ion channels (KCNH2), while undifferentiated hiPSCs express the pluripotency markers OCT4 and NANOG. **(C)** Extracellular recordings and field-potential duration (FPD) measurements from healthy hiPSC-derived cardiac tissue (control) (top) and hiPSC-derived cardiac tissue suffering LQTS. FPD was significantly longer in cardiac tissues derived from LQTS hiPSC-CMs in comparison to the control. **(D)** LQTS cardiac tissue from hiPSC-CMs showed marked arrhythmogenicity. Arrows identify single and multiple premature beats (Itzhaki et al., 2011).

As a consequence of the key role of ECM architecture on heart development and function, the ideal *in vitro* cardiac tissue model should accurately reproduce heart 3D anisotropic structure and vasculature in both healthy and pathological state, control and properly guide cell–cell and cell–ECM interactions and regulate cell fate and functions (Mathur et al., 2016). In pathological conditions, after a myocardial infarction or an ischemia, as well as with aging, the myocardium undergoes a remodeling process characterized by loss of functional cardiomyocytes, hypertrophy of the remaining cells and fibrosis, that result in a progressive alteration of heart mechanical properties (i.e., stiffness increase) and loss of function (Brower et al., 2006; Kwak, 2013). Consequently, an *in vitro* model of pathological cardiac tissue should accurately recapitulate cardiac changes in terms of structure, mechanical properties, cell density, and functions. Such an approach is essential in the design of suitable *in vitro* models for (i) drug development and testing (cardiotoxicity is a major cause of withdrawal of newly commercialized drugs) (Ferri et al., 2013) and (ii) the investigation of disease onset and progression. Both animal models and 2D cell cultures fail to accurately and fully reproduce human physiology in healthy and pathological conditions, as well as in young and aged states. For example, human CMs show significantly different electrophysiological properties with respect to the murine ones: mice heart rate at rest is about 10 times that of humans, while QT segment in humans is about four times that of mice (Passier et al., 2008; Vunjak Novakovic et al., 2014; Benam et al., 2015). As a consequence of the multifactorial nature of cardiovascular diseases, a different animal model should be used depending on the investigated pathology [e.g., porcine is a valid model of atherosclerosis and restenosis since it develops lesions more similar to human disease (Zaragoza et al., 2011; Leong et al., 2015)]. An appropriate selection of the model would be essential to avoid failing of many research findings upon translation to humans. On the other hand, traditional 2D *in vitro* cell cultures do not properly replicate the complexity of the *in vivo* environment and fail to maintain cardiac cells in culture for a long time, thus limiting the possibility of long-term studies. In this context, bioengineered healthy or pathologic heart tissue constructs can (i) exhibit genotypic and phenotypic properties more similar to native environment, (ii) reproduce cardiac tissue architecture, (iii) promote cell–cell and cell–ECM interactions, stimulating the formation of gap junctions, (iv) favor tissue maturation and the expression of a phenotypic profile similar to that of mature cardiomyocytes, and (v) allow accurate functional parameters measurements (Benam et al., 2015). In the design of a suitable and functional *in vitro* cardiac model, the first issue researcher’s interface with is the selection of the optimal source of beating CMs. Early developed heart models were based on immortalized or primary cells extracted from several species, such as chickens, mice, and rats, that did not accurately reproduce the physiology of the human heart, as previously mentioned, and failed in the reproduction of several pathological conditions, such as reduced conductivity, fibrosis, and scar formation (Harding et al., 2007). For these reasons, they were soon replaced by CMs derived from cardiac progenitor cells (CPCs), human ESCs, and hiPSCs that better recapitulate human physiology and pathophysiology (Benam et al., 2015; Mathur et al., 2016). Recent advancements in genome-editing technologies have opened the way to the possibility to engineer hiPSC genome by introducing the gene mutations associated with a specific heart disease, thus allowing the generation of pathological CMs to be used for disease modeling (Wang et al., 2012; Musunuru, 2013; Miyaoka et al., 2014). Do date, hiPSC-derived CMs have been successfully employed to model several rhythm-associated diseases [e.g., long QT syndrome (LQTS) and polymorphic ventricular tachycardia] and dilated and hypertrophic cardiomyopathies, which were thus thoroughly investigated at the cellular level (Morita et al., 2005; Moretti et al., 2010; Itzhaki et al., 2011; Jung et al., 2012; Sun et al., 2012) (Figure 3). Moreover, hiPSCs open the way to the development of patient-specific *in vitro* models that could be exploited in the future to better understand disease onset and progression, and specially to test the effects of drugs and therapies on each patient. Nevertheless, an important issue still needs to be addressed: CMs derived from stem cells are usually small in size and immature, showing a gene expression profile more similar to fetal CMs than adult cardiac muscle cells, and exhibit reduced contractility (Itzhaki et al., 2011). Since several topographical, electrical, mechanical, biochemical, and cellular cues actively contribute to heart development and CM maturation *in vivo*, efforts are currently directed to the exploitation of engineering methods (e.g., morphological and superficial cues, electrical, and mechanical stimulation) to induce stem cell-derived CMs maturation. As an example, properly surface-patterned substrates have been recently coupled with biochemical cues to enhance maturation of hiPSC-derived CMs (Ribeiro et al., 2015) and design a 3D beating human cardiac micro-chamber mimicking the developing human heart (Ma et al., 2015).

Two-dimensional *in vitro* models based on cell seeding on substrate surfaces functionalized with cardiac ECM proteins (e.g., laminin, fibronectin), or microfabricated according to well-defined anisotropic patterns successfully mimic CMs organization in the native cardiac tissue and recapitulate essential physiological and pathological properties of cardiac muscle cells (Simpson et al., 1994; Bursac et al., 2002; McDevitt et al., 2002; Motlagh et al., 2003; Camelliti et al., 2006; Cimetta et al., 2009; Kim et al., 2010; Chen et al., 2011; Luna et al., 2011; Wang et al., 2011a; Annabi et al., 2013; Ma et al., 2013). For instance, aligned murine CMs showed calcium handling, action potentials and conductivity more similar to adult mouse heart, with respect to the same cells cultivated on randomly oriented substrates (Thomas et al., 2000; Pong et al., 2011). The capability of such models to recapitulate the spatial heterogeneity and conductivity of cardiac tissue was successfully exploited to study pathologies of the electrical conduction system of the myocardium (Thomas et al., 2003; Beauchamp et al., 2004, 2012).

The same approach was exploited to design a model of the border zone (the interface between healthy cardiac tissue and an infarcted area) (Chang et al., 2009). *In vivo*, the border zone shows a non-uniform anisotropic structure resulting from the remodeling cascade activated by a myocardial infarction, which makes this tissue easily susceptible to arrhythmias. The developed model was based on human skeletal myotubes (simulating the typical fibrosis of the border zone) cocultured with neonatal rat ventricular CMs (recapitulating the non-uniform anisotropic architecture of the border zone) on fibronectin-coated coverslips. This system successfully modeled the onset of reentrant arrhythmias in the border zone, allowing the study of the effects of several drugs on this pathology and the explanation of the scarce effects of sodium channel blockers. However, *in vitro* 2D models fail to completely reproduce the mechanical contraction and architecture typical of the heart, thus hindering the ability of cells to interact and exert forces on each other. With the final aim of overcoming these drawbacks, several research groups designed 3D models recapitulating both healthy and pathological cardiac tissue. Scaffolds designed and fabricated for such applications should be biocompatible, exhibit a 3D structure with interconnected pores, which promote cell homing, nutrient and oxygen supply and waste removal, and reproduce both the structural and mechanical properties of the native cardiac tissue (Silvestri et al., 2013; Boffito et al., 2014). The literature reports on the fabrication of 3D scaffolds based on both natural and synthetic polymers and their blends by either conventional or advanced technologies. A widely investigated technology consists in cardiomyocyte loading into biodegradable natural polymers, e.g., collagen, Matrigel and fibrin, subjected to polymerization according to well-defined geometries (rings, cylinders, plates). Matrigel was often used to enhance cell viability and adhesion due to its composition rich in growth factors and ECM components (Mathur et al., 2016). Moreover, it was successfully blended with other natural polymers, such as fibrinogen and thrombin, or collagen type I, and seeded with neonatal rat CMs or stem cell-derived CMs to design models for drug screening (Hansen et al., 2010; Schaaf et al., 2011) or for the investigation of dilated cardiomyopathy and arrhythmogenesis (Thavandiran et al., 2013; Turnbull et al., 2014; Hinson et al., 2015). 3D cardiac tissue models were also successfully developed by culturing CMs derived from ESCs and CPCs on a fibrin-based hydrogel (Liau et al., 2011; Zhang et al., 2013). The diabetic myocardium was successfully recapitulated by seeding neonatal rat CMs on a collagen-based scaffold (Gelfoam^®^) (Song et al., 2011). The developed substrates were cultured in four different situations: normal glucose without or with the addition of insulin (N and NI, respectively), and high glucose without or with the addition of insulin (H and HI, respectively). Results demonstrated that, in diabetic conditions (i.e., H), the gene expression in the bioengineered constructs was similar to that observed in animal models, and accompanied by contractile dysfunctions and reduced electrical excitability. Insulin administration enhanced cell viability, contractility and normalized gene expression in both NI and HI models. On the other hand, administration of anti-diabetic drugs showed anti-apoptotic effects and enhanced excitability in bioengineered constructs cultured according to H conditions, but did not affect gene expression. Ring-shaped bioengineered cardiac tissues were developed to study ischemia/reperfusion conditions *in vitro* (Katare et al., 2010). Recapitulation of ischemic conditions (1% O_2_ for 6 h) induced the onset of conductive system defects, connexin-43 (the main cardiac connexin found in the gap junctions) dephosphorylation and the down-regulation of cell survival associated proteins, similarly to *in vivo* observations in humans (Ando et al., 2005). Such ischemic conditions turned out inhibited by treating the models with cardioprotective drugs, e.g., cyclosporine and acetylcholine (Ando et al., 2005). Synthetic polymers are promising alternatives to ECM-derived ones, due to their high versatility, repeatability and controlled composition. The literature reports the exploitation of several synthetic polymers for the design of both cardiac patches and *in vitro* models. The most suitable synthetic polymers for such applications are elastomers, e.g., poly(ester urethane)s (Guan et al., 2002, 2008; Sartori et al., 2013; Chiono et al., 2014; Silvestri et al., 2014; Boffito et al., 2015, 2016; Tonda-Turo et al., 2016), poly(glycerol sebacate) (Chen et al., 2008; Engelmayr et al., 2008; Guillemette et al., 2010; Ravichandran et al., 2011), and poly[(1,8-octanediol)-co-(citric acid)] (POC) (Hidalgo-Bastida et al., 2007; Prabhakaran et al., 2012), which can be easily processed by both conventional and non-conventional techniques. Bursacet al. successfully combined a 3D polyester-based porous scaffold seeded with neonatal rat-derived cardiac muscle cells with dynamic cell culture in a bioreactor to design a bioengineered tissue showing cardiac-specific structure and electrophysiology that make it suitable for *in vitro* studies of impulse propagation (Bursac et al., 2002). A model of LQTS was successfully developed by Ma and colleagues by seeding CMs derived from iPSCs from pathological patients on anisotropic scaffolds produced by two-photon initiated polymerization (Ma et al., 2014). Healthy cardiac tissue models were also fabricated by seeding iPSC-derived CMs isolated from healthy individuals. The models were validated by assessing their differences in terms of contractility, QT segment duration, tissue structure and response to several molecules, e.g., caffeine, nifedipine, and propranolol.

Aratyn-Schaus et al. recently developed an *in vitro* model of cardiac cell therapy to test the hypothesis that newly formed cardiomyocytes show a weak contractile strength that hampers stress transmission at the junction with native myocytes (Aratyn-Schaus et al., 2016). To this aim, two cell microtissues were designed by seeding mouse CMs, recapitulating native myocardium, and CMs derived from iPSCs and ESCs, modeling newly formed cells, on soft gels coated with fibronectin, according to a well-defined pattern, mimicking the striated structure and mechanical properties of the heart. The mechanical coupling between the two microtissues was thoroughly studied, demonstrating that newly formed CMs couple with native cells to support synchronous contraction, but the reduced force transmission between them may hamper the complete recovery of contractility. In 2017, the design of cardiac microtissues has been further enhanced by Giacomelli and coworkers that have recently developed a human-derived model recapitulating the cardiomyocyte–endothelium crosstalk, which is responsible for the regulation of heart dimension, oxygen supply, and growth factor secretion and has poorly been considered so far in both cardiac patch and engineered model design (Giacomelli et al., 2017).

A 3D scaffold-free cardiac tissue model based on multiple cell type coculture has been recently created for the first time by Rogozhnikov et al. by developing a strategy based on the combination of liposome fusion, bio-orthogonal chemistry, and cell surface engineering, with the ability to trigger and guide the self-assembly of three different cell types (cardiomyocytes, endothelial cells and cardiac fibroblasts) into a functional 3D cardiac tissue (Rogozhnikov et al., 2016). Such an approach could open the way to a new era in cardiac TE, allowing the development of high cell density constructs showing spontaneous synchronous beating throughout the entire matrix (without the application of electrical cues) and efficient cell–cell and cell–ECM crosstalk.

## Pancreas *In Vitro* Models

The pancreas is an organ characterized by two distinct functional portions: the exocrine and the endocrine pancreas. The functional unit of the exocrine pancreas is the pancreatic acinar cell, which has the main functions of synthesis, storage and secretion of digestive enzymes. These enzymes are normally activated in the duodenum, meanwhile acute pancreatitis occurs when they are prematurely activated within the pancreatic acinar cell. The endocrine portion of the pancreas is constituted by small clusters of cells called islets of Langerhans. Pancreatic islets contain five cell types: alpha cells (α cells) secrete the hormone glucagon; beta cells (β cells), the most abundant cells in the islets, produce insulin; delta cells (δ cells) secrete the hormone somatostatin; epsilon cells (ε cells) secrete ghrelin, and PP cells secrete pancreatic polypeptides. Moreover, several minor hormones are synthetized from pancreatic islet cells. Hormone secretion is modulated by nervous signals and, thanks to a high vascularization, hormones secreted by islets have ready access to the circulation (McManus and Mitchell, 2014).

Type I diabetes is a chronic pathological condition in which a cell-mediated autoimmune destruction of pancreatic β cells usually leads to a deficiency in insulin release (Devendra et al., 2004; Daneman, 2006; American Diabetes Association, 2010). Type II diabetes is the most diffused form of diabetes (Moller, 2001; Stumvoll et al., 2005; Daneman, 2006; Nanji and Shapiro, 2006), characterized by disorders of insulin action, due to insulin resistance, and abnormalities in insulin secretion by β cells (Assal and Groop, 1999; Stumvoll et al., 2005). Due to the high relevance of these diseases (Nanji and Shapiro, 2006), pancreas studies have been mostly focused on the islets of Langerhans. Moreover, islets are a key focus of type II diabetic drug efficacy testing (Li et al., 2013a).

Mice constitute the most investigated animal model in pancreas research. However, there are differences in Langerhans islets cell population of mice and human: the ratio β cells/α cells is higher in mice than in humans, meanwhile δ and PP cells are double in humans with respect to mice (Dolenšek et al., 2015). Also the architectures of human and mice pancreas present some differences, promoting heterologous contacts between α and β cells in humans, which result in a higher sensitivity to glucose compared to mouse islets (Dolenšek et al., 2015). In 2009, Kim and colleagues studies (Kharouta et al., 2009; Kim et al., 2009) showed that mice present human-type islets when subjected to pathologic conditions characterized by an increased demand for insulin, such as inflammation, obesity, diabetes, and pregnancy. These species differences have raised concerns regarding the use of mice prototypic islets, highlighting the need to develop more realistic islet models.

The first challenge in engineering pancreatic islets is the isolation and purification of viable and functional islets. It has been shown that following islet isolation and purification, apoptotic cell death occurs, involving mainly β cells (Rosenberg et al., 1999). This response is mainly due to the loss of cell–cell communication (Ilieva et al., 1999), cell–matrix interactions and the disruption of the peri-insular basement membrane (Rosenberg et al., 1999; Wang and Rosenberg, 1999), a critical component of the pancreatic ECM, mainly composed of type IV collagen, laminin, and fibronectin (Wang and Rosenberg, 1999). Moreover, when cultured *in vitro*, islet cells undergo necrotic cell death predominantly in the islet core, as a consequence of inadequate oxygen supply due to the high metabolic demands and islet size (Andersson, 1978; Ilieva et al., 1999).

In order to reach a successful strategy to preserve islet cell survival and functions, several studies have focused on restoring the ECM environment. In particular, it has been demonstrated the positive response of pancreatic islets to collagen I/collagen IV mixture (Nagata et al., 2001), laminin (Hammar et al., 2005), and peptide epitopes (e.g., RGD sequence of fibronectin) (Pinkse et al., 2006), mimicking fundamentals components of the peri-insular basement membrane of pancreatic ECM.

Three-dimensional cultures are preferred to 2D, since β cells easily lose viability and the ability to secrete insulin when cultured on 2D traditional systems (Benam et al., 2015). From this point of view, tissue-engineered scaffolds represent a valid alternative to effectively support pancreatic cell culture and secretion of hormones and polypeptides, while bioreactors are useful tools to provide the required perfusion conditions to prevent *in vitro* cell necrosis (Bentsi-Barnes et al., 2011; Li et al., 2013b).

Synthetic polymeric scaffolds have been extensively studied for islet culture and transplantation. In particular, polyester scaffolds were fabricated from lactide and glycolide monomers and copolymers, using different fabrication techniques (Blomeier et al., 2006; Chun et al., 2008; Mao et al., 2009).

Blomeier and colleagues fabricated macroporous biodegradable scaffolds (pore size in the range of 250 to 400 µm) from copolymers of lactide and glycolide (PLGA) using a gas foaming/particulate leaching process, which improved islet function *in vivo* (Blomeier et al., 2006). Mao et al. developed a construct constituted of PLGA scaffolds (pore size between 100 and 300 µm) seeded with islet-like cells, which transplanted *in vivo* in diabetic immunodeficient mice led to correction of blood glucose levels (Mao et al., 2009). In another study, rat islet cells were seeded on porous polyglycolic acid (PGA) fiber scaffold (Synthecon Inc.) previously coated with poly-l-lysine (PLL) in order to improve cell adhesion (Chun et al., 2008). In contrast to the scaffold-free control group, islet cells cultured on scaffolds exhibited improved morphology, viability, and increased insulin secretion.

Functionalization of synthetic polymeric scaffolds with bioactive molecules, such as ECM components, has been proposed to mimic the ECM environment. Salvay et al. (2008) demonstrated the successful use of PLGA scaffolds produced by gas foaming process and coated with collagen IV, fibronectin, and laminin for culturing and transplant mouse islets. Those scaffolds showed good viability, insulin production and vessel density within the transplanted islets.

Although synthetic polymeric scaffolds have been successfully employed according to the classic TE approach, they are not adequate to replicate pancreas mechanical properties, which play a key role in the design of valuable tissue and organ models. In fact, the pancreas is a non-linear viscoelastic soft tissue. The shear stiffness of healthy pancreatic tissue was found to be in the range of 1–2 kPa (Wex et al., 2015), whereas the stiffness of polyesters is higher, namely elastic modulus is generally in the range of 6–7 GPa for polyglycolide (Van de Velde and Kiekens, 2002) and 2–3 GPa in the case of polylactic acid and poly(lactic-co-glycolic) acid (Gentile et al., 2014). On the other hand, porous scaffolds used in the previously mentioned studies did not resemble pancreas morphology and a justification for the architecture selection is not generally reported.

It should be observed that further investigations are necessary to understand how pathologic conditions affect the mechanical properties of pancreatic tissue, such as in chronic pancreatitis (Janssen et al., 2007). In the diagnosis of chronic pancreatitis, it is presumed that pancreatic shear stiffness is proportional to the degree of fibrosis (Ziol et al., 2005; Georges et al., 2007). There is a lack of studies regarding the mechanical characterization of the scaffolds proposed, which is particularly important to elucidate the different behavior of healthy and pathological tissues. Therefore, pathological and physiological models may be achieved taking into account this consideration.

Synthetic and natural-derived hydrogels have been demonstrated to be good candidates as scaffold materials for pancreatic TE because of their high water content, structural and mechanical similarities with the native ECM (Cushing and Anseth, 2007), and high permeability for low molecular weight nutrients, metabolites and hormones, which is a key point in β cell survival.

**Figure 4:**
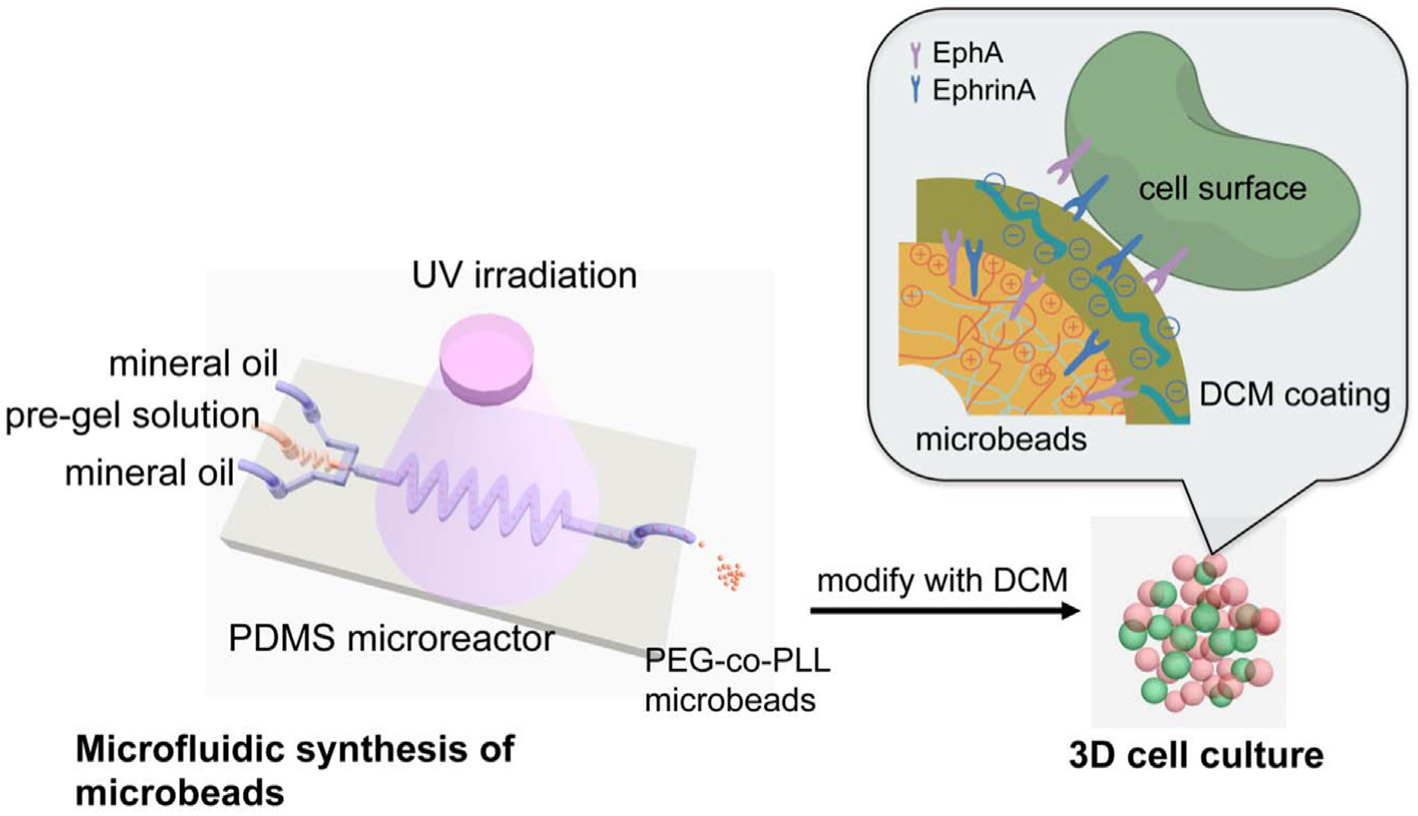
Schematic reproduction of the microbeads’ microfluidic synthesis, and three-dimensional (3D) cell culture. The microbeads were made from cross-linked poly(ethylene glycol)-co-poly-l-lysine (PEG-co-PLL) hydrogels modified with the cell surface receptor and its membrane-bound ligand pair, EphA/EphrinA, and coated with pancreatic tissue-specific extracellular matrix components derived from rat pancreatic decellularized matrix. The microbeads (in red) were then cultured in direct contact with β cells (in green) mimicking the cell–cell interaction that cell experiences *in vivo*. Reprinted with permission from Li et al. (2013a).

Li et al. (2013a) developed an *in vitro* model of 3D pancreatic islet microenvironment to study the stability and functions of β cells. In this research work, microbeads made from cross-linked poly(ethylene glycol)-co-poly-l-lysine (PEG-co-PLL) hydrogels were developed to act as “synthetic neighbors” of the β cells, aiming to reproduce the high cellular density environment of pancreatic islets. The copolymerization of PLL with PEG was designed to introduce positive charges on the gel surface, allowing the absorption of ECM components derived from rat pancreatic decellularized matrix and inducing cell–ECM interactions. In order to mimic the *in vivo* cell–cell interactions, microbead surface was modified with the cell surface receptor and its membrane-bound ligand pair EphA/EphrinA (Figure 4). In fact, in the pancreatic islets, β cells communicate via EphA receptors and EphrinA ligands (Konstantinova et al., 2007). This 3D microenvironment was able to mimic both native cell–cell and cell–ECM interactions, enhancing β cell viability (up to 21 days) and consequently supporting insulin production.

Among natural-derived hydrogels, alginate has been successfully used in the classic approach of pancreatic TE, where microencapsulation of the islets is performed to preserve them from immune mediated destruction after transplantation (Lim and Sun, 1980; Korbutt et al., 2004). Thus, alginate hydrogels could represent a valid scaffold for the development of pancreatic *in vitro* models. However, the major limitation of alginate microbeads is the high permeability to a range of small molecules, which can damage or destroy the encapsulated islets (Van Schilfgaarde and De Vos, 1999). To overcome these problems a perm-selective coating with PLL or Poly-l-Ornithine has been proposed, since these polyamino acids firmly bind to alginate, thereby restricting the permeability of alginate-based microcapsules (Thu et al., 1996; Darrabie et al., 2005). In contrast, the capsule must be permeable to nutrients, metabolites and hormones to allow islet survival. It was observed that a reduction in capsule size improves the diffusion of nutrients to the islets. Omer et al. demonstrated that alginate capsules with a diameter of 600 ± 100 µm showed improved stability *in vivo* over larger capsules with diameters of 1000 ± 100 µm (Omer et al., 2005). Nevertheless, with reduction in capsule size an increase in the number of capsules containing protruding islets was observed, leading to a higher number of capsules affected by an inflammatory response. Decreasing the islet density in alginate can solve this problem. Not only the size but also the morphology of the microcapsules is an important parameter: spherical microcapsules are necessary for long-term functions (Hobbs et al., 2001).

In order to overcome problems related to the supply of oxygen and nutrients, Li et al. proposed an *in vitro* perfused 3D model for diabetic drug efficacy tests (Li et al., 2013a), able to mimic the *in vivo* perfusion conditions of pancreatic islets (Menger et al., 1994). Specifically, rat islets were encapsulated in ultrapure alginate and cultured *in vitro* in a commercial micro-bioreactor system (TissueFlex^®^). The system supported islet viability and functions *in vitro* over a 7-day culture period. The model displayed a high sensitivity in responding to two typical anti-diabetic drugs (tolbutamide and GLP-1) and drug dosages over conventional 2D and 3D static models.

Bioreactors have shown to be important in the modeling of pancreatic islets environment and in the understanding of the effects of dynamic culture conditions on β cell performance. Hou et al. cultured rat pancreatic islets in stasis and simulated microgravity, in the presence or absence of a PGA fibrous scaffold (Hou et al., 2009). The simulated microgravity was achieved through a rotary cell culture system (Cameron et al., 2001; Murray et al., 2005), which allowed high mass transfer of nutrients by maintaining normal morphology of the islets. After 5 days in culture in this bioreactor, the islet grafts were transplanted into leg muscles of diabetic rats to observe their functions and morphology. The results demonstrated that islets cultured under dynamic conditions in the scaffolds exhibited better viability and insulin production compared to those cultured in static condition, confirming the importance of both scaffolds and dynamic culture in the mimesis of the native environment and the maintenance of cell viability and functions. This approach can be translated to the design of an *in vitro* functional islet model.

Pancreas TE is mainly focused on the encapsulation of Langerhans islets for further transplantation. The requisites that should be met in that case are different from those required in the development of an *in vitro* model of the islets themselves. However, there are some common requisites, such as the challenging maintenance of the islet cell viability and functions after isolation. The research works surveyed in this review reported a progress in the design of biomimetic constructs able to support both cell survival and functions in culture; however, more engineered systems are needed to develop valuable pancreas models. Such a goal is challenging, due to the complexity of this organ. Forthcoming pancreatic models need to mimic the native tissue in all the mechanical, topographical and chemical aspects, as well as in the set of physiological cues that characterize the complex pancreatic environment. The models developed for the pancreas are few, indicating that the design of a model for such complicate organ requires further efforts and a closer collaboration between different fields of research.

## *In Vitro* Liver Models

The liver is the largest visceral organ in humans, playing a wide and complex array of vital functions, ranging from metabolic and regulatory activities to protein synthesis and organism defense processes (Mazzoleni and Steimberg, 2012). The liver is characterized by a complex array of vasculature, endothelial cells and parenchymal cells (hepatocytes, hepatocyte precursors, stellate cells, epithelial cells and fibroblasts) (Lorenzini and Andreone, 2007; Kazemnejad, 2009). Hepatocytes are the leading hepatic parenchymal cell type in terms of both mass (about 60% of liver cells) and number of functions carried out. Liver ECM plays a pivotal role on hepatocyte viability, proliferation, migration and functions (Mazzoleni and Steimberg, 2012). In fact, as a consequence of their structural and functional polarization, hepatocytes need well-defined cell–cell and cell–ECM interactions to remain viable, proliferate and exert their activities. From a structural point of view, the liver is characterized by a complex, highly organized architecture composed of a tessellating system of hexagonal constructs (lobules), fundamental for maintaining hepatic functions (Patzer and Gerlach, 2011; Mazzoleni and Steimberg, 2012).

In pathological conditions of fibrosis, liver architecture is distorted because of ECM proteins accumulation and the formation of fibrous scar tissue, which induce an increase in tissue stiffness (Bataller and Brenner, 2005). Advanced fibrosis results in cirrhosis that causes dysfunctions and is responsible for hepatic insufficiency (Bataller and Brenner, 2005).

At present, the procedure leading to the clinical application of newly developed drugs requires more than 10 years and a high economical investment (about one billion euros) (Adams and Brantner, 2006). Moreover, the majority of the drugs reaching phase III clinical trials shows hepatotoxicity, which hampers their approval for administration in humans, and about one third of approved drugs are withdrawn from the market for unpredicted liver toxicity through currently used experimental setups (O’Brien et al., 2004; Whitebread et al., 2005; Mazzoleni and Steimberg, 2012). Currently, no animal model fully recapitulates all the hepatic and extrahepatic features of healthy and pathological liver: first, some hepatic diseases do not exist in rodents, and second, animals can show higher or lower susceptibility to drugs compared to humans (Delire et al., 2015). A wide variability is usually observed between humans and animal models in terms of drug pathogenicity, time of action and effects (Starkel and Leclercq, 2011). In this context, there is increasing interest in developing more reliable *in vitro* liver models, able to imitate liver functions in pharmacokinetics, thus allowing a more accurate drug testing as well as compliance to 3Rs principle. At the same time, a thorough investigation of hepatic functions and pathologies (e.g., fibrosis, cirrhosis, fatty liver) and the molecular mechanisms at their basis could contribute to a better characterization and understanding of the role of this organ in human homeostasis and metabolism, as well as disease onset and progression.

The simplest *in vitro* liver models consisting of hepatic sub-cellular fractions (e.g., single enzymes, cytosolic factions, mixed fractions) are easy to use and suitable for the investigation of single metabolic functions (Mae et al., 2000; Clarke and Jeffrey, 2001; Rawden et al., 2005; Boelsterli and Lim, 2007). However, they cannot be exploited to assess the influence of other parameters/functions on the modeled metabolic pathway and fail in the accurate reproduction of liver complex structure, which strongly influences its functions. 2D liver tissue models comprising monolayer and collagen-sandwich cultures are more reliable than sub-cellular fractions and allow long-term cell culture. Nevertheless, they fail in the mimesis of cell–cell and cell–ECM interactions, essential for hepatocyte viability and functions, resulting in cell dedifferentiation and loss of the majority of their phenotypic properties, including their drug-metabolizing capacity, thus hampering their application in drug testing (Mazzoleni and Steimberg, 2012; Mueller et al., 2013). Primary human hepatocytes are the gold standard in the design of *in vitro* models reproducing human liver functions for the investigation of drug metabolism and toxicity, as in the study of disease onset, progression and response to new therapies (Gómez-Lechón et al., 2007; Mazzoleni and Steimberg, 2012; Mueller et al., 2013; Willebrords et al., 2015). However, the capability to maintain liver native functions for only few days (about three days) under conventional cell culture conditions, together with tissue shortage and its high variability, have limited the establishment of 2D primary hepatocyte cell culture and consequently the use of these cells in the development of liver models (Guillouzo and Guguen-Guillouzo, 2008; Mazzoleni and Steimberg, 2012; Mueller et al., 2013; Soldatow et al., 2013). Primary rodent hepatocytes have been widely exploited in drug testing and drug-drug interaction studies, but the translation of the obtained results to humans is made difficult by differences in metabolism among the two species (Uehara et al., 2008; Lauer et al., 2009). On the other hand, human immortalized hepatic cancer cell lines, such as HepG2, show unlimited availability and maintain certain liver functions, e.g., albumin production (Mueller et al., 2013). However, they do not exhibit drug-metabolizing capacities, which can cause inaccurate drug toxicity testing. In several studies, immortalized hepatic cells turned out suitable models for parent compound toxicity studies and in the assessment of cell polarity and chemotherapy resistance (Hoekstra et al., 1999; Niklas et al., 2009; Noor et al., 2009; Mueller et al., 2012). Hepatocytes derived from stem cells (embryonic- and adult tissue-derived stem cells) exhibit promising liver-specific features, such as the expression of cytochrome P450 (PYP450) enzymes (enzymes involved in the metabolism of many molecules) and the formation of structures similar to the bile canaliculi. Moreover, they provide a highly available hepatocyte source from different donors, thus enhancing reproducibility and allowing the study of individual-specific drug toxicity. However, the secretion of liver-specific proteins and enzymes, as well as the expression of CYP450 enzymes and membrane transporters, need to be comparable to those of native hepatocytes (Mueller et al., 2013; Soldatow et al., 2013; Davidson et al., 2015). 3D liver models accurately replicating liver tissue in terms of architecture and functions are gaining more interest as efficient systems ensuring long-term cell viability and functions and allowing the study of drug metabolism, liver adverse effects and repeated dose testing (Vinci et al., 2010; Mueller et al., 2013; Soldatow et al., 2013). Perfused livers and liver slices show the advantage of retaining the 3D architecture, cell–cell and cell–ECM interactions of the native organ, but they do not allow long-term studies since necrosis occurs within 48–72 h and metabolic enzymes levels decrease in 6–72 hours (Mueller et al., 2013; Soldatow et al., 2013). Therefore, the use of biomaterial-based 3D liver tissue models is a rapidly expanding field, devoted to the design of novel smart constructs, accurately mimicking liver tissue in its healthy and pathological state, from a morphological, mechanical and biochemical point of view. The literature reports several *in vitro* 3D liver models based on native or stem cell-derived liver cells, cultured under static or dynamic conditions in ECM-based scaffolds (decellularized liver), 3D porous scaffolds/hydrogels based on natural or synthetic polymers. For instance, Mazza et al. have recently reported a successful human liver decellularization protocol, with preservation of the native honeycomb architecture of connective tissue fibers and essential ECM proteins (collagen type I and IV, fibronectin). The procedure was followed by repopulation with human liver-derived cells, thus confirming that the optimized decellularization procedure did not damage the liver tissue features, essential for cell homing, migration and proliferation (Mazza et al., 2015). Similar results were also reported by Uygun and colleagues, which also demonstrated that the preservation of the native vasculature results in enhanced cell engraftment, survival and maintenance of hepatocyte functions (albumin secretion, urea synthesis and cytochrome P450 similar to normal liver) (Uygun et al., 2010). The 3D structure resulting from decellularization is also an efficient substrate for stem cell differentiation: Wang et al. reported hepatic stem cell differentiation into parenchymal cells, showing mature and stable phenotype for more than 2 months (Wang et al., 2011b).

Among synthetic polymers, biocompatible and biodegradable polyesters, e.g., PLGA, poly(l-lactic acid) (PLLA), PGA and poly(ε-caprolactone) (PCL), have been widely investigated in hepatic TE. PLGA- and PLLA-based 3D porous scaffolds showed the ability to favor primary hepatocyte adhesion, proliferation and maturation (Kim et al., 1998; Hasirci et al., 2001; Huang et al., 2006), as well as ESC differentiation toward the hepatocyte lineage (Liu et al., 2009). 3D nanofibrous scaffolds produced by electrospinning were identified as promising substrates for stem cell differentiation toward hepatocytes (Hashemi et al., 2008; Kazemnejad et al., 2009; Farzaneh et al., 2010; Piryaei et al., 2011). For instance, several studies reported the hepatic differentiation of bone marrow-derived MSCs (Kazemnejad et al., 2008, 2009; Piryaei et al., 2011), human ESCs (Farzaneh et al., 2010) and cord blood-derived stem cells (Hashemi et al., 2008) cultivated on electrospun matrices. Moreover, Vinci et al. have recently designed an *in vitro* liver model based on a multi-layer PLGA-based scaffold fabricated by pressure assisted microsyringe using a hexagonal repetitive unit mimicking hepatic lobules (Vinci et al., 2010). This study demonstrated that cell density and cell–cell interactions are strongly influenced by substrate architecture, while cell metabolism is mainly regulated by nutrient supply and interstitial-like flow (Vinci et al., 2010). Indeed, albumin and urea production rates turned out greatly augmented during dynamic cell culture in a low-shear, high-flow bioreactor. The presence of integrin-binding sites and specific sequences in natural polymers can be exploited to activate the desired cell behavior and signaling cascades. Dvir-Ginzberg and colleagues reported the capability of alginate scaffolds to maintain hepatocyte functions during 1-week culture time (Dvir-Ginzberg et al., 2003) and allow liver cell reorganization into spheroids and their development into hepatic functional tissue after 6 weeks (Dvir-Ginzberg et al., 2008). Furthermore, alginate scaffolds showed ability to induce hepatic differentiation of bone marrow-derived stem cells in the presence of specific growth factors (Lin et al., 2010). Hyaluronate-based scaffolds promoted hepatic functions of liver cells as a consequence of the signaling pathways activated by cell binding to the material (Zavan et al., 2005; Katsuda et al., 2010), since hepatocytes have specific binding sites for hyaluronate (Frost et al., 1990). Similarly, chitosan properly modified with specific polysaccharide residues can bind liver cells, thus improving their functions and metabolic activity (Li et al., 2003; Feng et al., 2009). Moreover, doping natural polymer-based scaffolds with conducting polymers seems to positively influence hepatocyte adhesion and proliferation, enhancing the electrical communication among cells (Rad et al., 2014).

**Figure 5:**
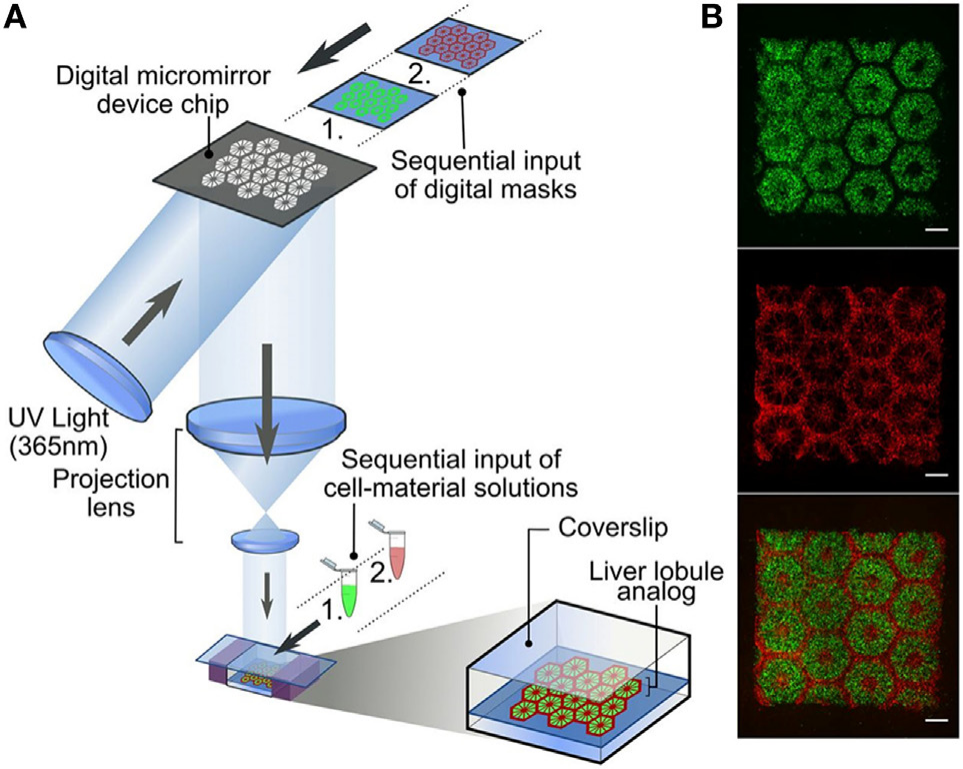
Three-dimensional (3D) printed hepatic lobule models. **(A)** 3D bioprinting of human iPSCs (hiPSC)-derived hepatocytes and supporting cells encapsulated in photocurable hydrogels [5%w/v gelatin methacrylate (GelMa) for hiPSC-derived hepatocytes to obtain, after photopolymerization a matrix with stiffness similar to the healthy liver tissue, a blend of glycidal methacrylate-hyaluronic acid and GelMa for supporting endothelial and mesenchymal cells to favor endothelial cell proliferation and support vascularization processes] was carried out in two steps according to well-defined patterns **(B)** to finely reproduce their localization in native lobules. In detail, hiPSC-derived hepatocytes were first patterned according to a digital mask with proper geometry, followed by the patterning of the supporting cells using a second digital mask (scale bars 500 µm, supporting cells and hiPSC-hepatocytes were fluorescently marked in red and green, respectively). Reprinted with permission from Ma et al. (2016).

Induced pluripotent stem cells have recently arisen as promising candidates for the design of patient-specific hepatic models. In detail, Ma et al. encapsulated hiPSC-derived hepatic cells and supporting endothelial and mesenchymal cells in gelatin-based hydrogels and 3D printed the resulting cellularized biomaterials according to predefined biomimetic patterns mimicking hepatic lobule structure (Figure 5) (Ma et al., 2016).

From a morphological point of view, hiPSC-derived hepatocytes, human vein endothelial cells and adipose tissue-derived stem cells were embedded in a 3D hexagonal structure, according to well-defined patterns finely mimicking their localization in the native lobules. On the other hand, from a functional point of view, high expression of liver-specific genes, increased metabolic activity, and enhanced cytochrome P450 induction were observed. Therefore, the designed 3D tri-culture model showed improved morphological, phenotypic and functional properties, thus opening the way to the clinical introduction of *in vitro* models for personalized *in vitro* drug screening and disease study.

## Discussion and Future Directions

Nowadays, TE approaches are widely investigated for the development of 3D *in vitro* models of healthy and pathological tissues and organs. The results summarized in this review demonstrate that the TE approach can be successfully employed in the development of 3D models of many human tissues and organs, such as bone, heart, pancreas, and liver. This interdisciplinary field is rapidly developing and advancing. However, despite the already published exciting results in the design, fabrication, and validation of organ/tissue models, there are still challenges that need to be addressed.

The main limitation deals with the identification of the proper cell sources for model design, and in particular with the difficulty to isolate human primary cells and culture them *in vitro* for long-term experiments, since primary cells show high sensitivity to culture conditions and progressive loss of differentiation potential after a low number of passages in culture. In the last decade, the most promising novelty in the cell biology field is the discovery of iPSCs. Reprogramming adult cells to embryonic-like states has innumerable potential applications in regenerative medicine and drug development. iPSC-related research fields are highly active and rapidly developing. iPSCs are interesting cell sources and represent a breakthrough that will ultimately open many new avenues, although many technical and basic science issues remain.

The immature phenotype of differentiated cells derived from progenitor cells (induced, embryonic and adult stem cells) makes them appropriate for neonatal tissue/organs or early-stages diseases models. Moreover, the properties of human-derived cells strongly depend on the tissue source, the patient age and health condition, the adopted isolation/purification technique as well as the applied differentiation protocol.

Three-dimensional cell surrounding environment exerts a synergistic role in guiding cell fate and behavior; therefore, a fine replication *in vitro* of the *in vivo* environment in terms of both architecture and mechanical properties is mandatory. Furthermore, the development of a biomimetic environment is a key aspect in the long-term culture of any type of cells. Such a goal is challenging, due to the complexity of human organs/tissues and the difficulty to mimic them at different aging and health stages in all the mechanical, topographical and chemical aspects, as well as in the set of physiological cues characteristic of their environment. In this scenario, bringing together new advances in material engineering, microfabrication techniques and microfluidics is gaining more and more importance.

The advancement in biomaterials science, including the design and development of new synthetic copolymers, ceramic and glass–ceramics, bioartificial blends of natural and synthetic materials, can be exploited to finely tune the chemical, thermal, mechanical and surface properties of the scaffold-forming materials (Baino and Vitale-Brovarone, 2011; Sionkowska, 2011; Sartori et al., 2013; Boffito et al., 2014, 2015; Silvestri et al., 2014; Baino et al., 2015; Chiono et al., 2016). The progress of these custom-made materials allows to accurately recapitulate the bulk properties of the native tissue at different health levels.

Furthermore, emergent advanced scaffold fabrication methods are gaining more and more interest as they allow the fabrication of more reproducible scaffolds with a highly controlled process. These include a good control of pore size and interconnection, which facilitates gas diffusion, nutrient supply and waste removal, leading to a degree of vascularization of the constructs similar to native tissues.

Specifically, techniques based on rapid prototyping, bioprinting, organ printing and bottom-up approaches are emerging as promising tools, with the potential to overcome the drawbacks of conventional approaches, and providing a step forward the clinical validation of 3D *in vitro* engineered tissue models as a consequence of their high versatility, temporal and spatial control (Elbert, 2011; Xu et al., 2011; Arslan-Yildiz et al., 2016). Finally, to increase the industrial scalability of the models and allow high-throughput applications, recent developments in cell biology, TE, microfluidics and biomaterials are now being integrated in microfluidically perfused organ-on-chip models (Baker, 2011; Huh et al., 2012, 2013; Esch et al., 2015).

Some relevant *in vitro* tissue models have recently started to appear in the literature, improving the confidence that, in the future, the design of 3D models of high quality and relevance can significantly reduce the number of animals used in research as well as the failure of drug-screening methodologies.

## Author Contributions

SC, MB, and SS contributed with conception and design of work and writing of the paper. All the authors agree on the final version to be published.

## Conflict of Interest Statement

The authors declare that the research was conducted in the absence of any commercial or financial relationships that could be construed as a potential conflict of interest.
